# Association of Residential Mobility Over the Life Course With Nonaffective Psychosis in 1.4 Million Young People in Sweden

**DOI:** 10.1001/jamapsychiatry.2018.2233

**Published:** 2018-08-22

**Authors:** Ceri Price, Christina Dalman, Stanley Zammit, James B. Kirkbride

**Affiliations:** 1 MRC Centre for Neuropsychiatric Genetics and Genomics, Division of Psychological Medicine and Clinical Neuroscience, Cardiff University, Cardiff, Wales; 2 Epidemiology of Mental Health, Department of Public Health Sciences, Karolinska Institutet, Stockholm, Sweden; 3 Centre for Academic Mental Health, Bristol Medical School, University of Bristol, Bristol, England; 4 PsyLife group, Division of Psychiatry, UCL, London, England

## Abstract

**Importance:**

Residential mobility (changing residence) during childhood and early adolescence is a possible risk factor for several adverse health outcomes, including psychotic disorders. However, it is unclear whether sensitive periods to residential mobility exist over the life course, including in adulthood, or if greater moving distances, which might disrupt social networks, are associated with a greater psychosis risk.

**Objective:**

To examine the association between residential mobility over the life course and the risk of nonaffective psychosis.

**Design, Setting, and Participants:**

This prospective cohort study included all people born in Sweden between January 1, 1982, and December 31, 1995, who were alive and resided in Sweden on their 16th birthday who were followed up until up to age 29 years (ending December 2011). Participants were followed until receiving a first diagnosis of an *International Statistical Classification of Diseases and Related Health Problems, Tenth Revision (ICD-10) *nonaffective psychotic disorder (F20-29), emigration, death, or the end of 2011, whichever was sooner. National register linkage provided exposure, outcome, and covariate data (complete data were available for 1 440 383 participants [97.8%]).

**Exposures:**

The exposures to distance moved and the number of residential moves were examined for participants at the following periods over the life course: 0 to 6 years, 7 to 15 years, 16 to 19 years, and 20 years and older.

**Results:**

This study included 1 440 383 participants, of whom 4537 (0.31%) had nonaffective psychotic disorder (median age, 20.9 [interquartile range, 19.0-23.3]). More frequent moves during childhood and adolescence were associated with an increased risk of nonaffective psychosis that showed dose-response associations independent of covariates. The most sensitive period of risk occurred during late adolescence; those who moved during each year between age 16 to 19 years had an increased adjusted hazard ratio (HR) of 1.99 (95% CI, 1.30-3.05) compared with those who never moved. One move during adulthood was not associated with psychosis risk (adjusted HR, 1.04; 95% CI, 0.94-1.14), but moving 4 or more times during adulthood was associated with increased risk (adjusted hazard ratio, 1.82; 95% CI, 1.51-2.23). Independently, moving greater distances before age 16 years was associated with an increased risk (adjusted HR, 1.11; 95% CI, 1.05-1.19), with evidence of a nonlinear threshold effect for moves longer than 30 km. The distance moved after age 20 years was associated with a decreased risk (adjusted HR, 0.67; 95% CI, 0.63-0.71).

**Conclusions and Relevance:**

Children and adolescents with less disruption in their residential environments are less likely to experience psychotic disorders in early adulthood. Moves that may necessitate changes in school and social networks were most strongly associated with future risk.

## Key Points

**Question:**

Is residential mobility over the life course associated with the subsequent risk of developing nonaffective psychotic disorders?

**Findings:**

In this prospective cohort study of 1.4 million young people living in Sweden, followed up each year from birth to age 29 years, more frequent moves during childhood and adolescence were associated with increased risk for nonaffective psychosis, peaking between age 16 to 19 years, with moves longer than 50 km independently associated with greater risk. There was less evidence that moving in adulthood increased risk, except among those who moved 4 or more times.

**Meaning:**

The risk of nonaffective psychosis in young people was associated with greater residential mobility during formative periods of childhood and adolescence, which is consistent with the possibility that the disruption of social networks, peer support, and identity formation are relevant to the etiology of psychosis.

## Teaser

This cohort study examines the association of residential mobility during childhood, adolescence, and adulthood with risk of developing nonaffective psychosis in young people in Sweden.

## Introduction

International migration is an established risk factor for psychosis,^1^ and the risk appears greatest for those migrating at earlier ages.^2,3^ Some studies have suggested that internal migration (known as residential mobility) may also increase psychosis risk.^4,5^ Evidence from Denmark has suggested that long-distance childhood residential mobility increases the subsequent risk of schizophrenia and other nonaffective psychoses,^4,5,6^ with some evidence of a stronger effect of residential instability during adolescence than in childhood.^4^ However, to our knowledge, none of the studies have examined the effect of residential mobility beyond midadolescence, which may be particularly important given that some have suggested that higher rates of psychotic disorders in more deprived, socially fragmented urban environments^7,8,9^ are a consequence of social drift during the prodromal phases of disorder, as people may move into cheaper, more socially isolated environments.^10^ Moreover, to date, the geographical distances people move have been crudely treated as moves between large administrative areas, potentially obscuring the nuanced effects of moving over smaller or larger geographical distances.

In this study, we used data from individuals within a large population-based cohort, whose residential moves over their entire early life course (up to age 29 years) could be identified to small area neighborhood resolution, to examine the risk of developing nonaffective psychotic disorders associated with residential mobility during childhood, adolescence, and early adulthood. We focused on nonaffective psychoses, given stronger evidence that these psychiatric disorders are more strongly associated with urbanicity and migration than other disorders, such as bipolar disorder or unipolar depression.^11,12^ Given previous evidence, we hypothesized that having more frequent residential moves in childhood, adolescence, and early adulthood would be associated with an increased psychosis risk and that this would be highest for individuals who moved during adolescence, which is a key period for social development.^4,13^ We also hypothesized that the risk would increase with greater geographical distances moved in childhood and adolescence but in a nonlinear fashion, representing a “threshold” effect at which most moves were likely to result in a breakup of social networks (eg, due to an enforced change of school).

## Methods

### Sample

We identified all individuals born in Sweden between January 1, 1982, and December 31, 1995, who resided in Sweden on their 16th birthday from the Total Population Register. This study received ethical approval through Psychiatry Sweden from the Stockholm Regional Ethical Review Board and consent was waived. Persons were followed up from their 16th birthday until receiving a first diagnosis of an *International Statistical Classification of Diseases and Related Health Problems, Tenth Revision (ICD-10)* nonaffective psychotic disorder, censorship due to emigration, death, or December 31, 2011, whichever was sooner. We excluded first-generation immigrants because information on their residential mobility before moving to Sweden was unavailable. From our initial cohort (N = 1 472 446), our final analytical sample included 1 440 383 participants with complete residential mobility data ( eMethods in the Supplement).

### Outcome Measure

Our main outcome measure was a clinical diagnosis of nonaffective psychotic disorder (*ICD-10*: F20-29), including schizophrenia (F20) and other nonaffective psychoses (F21-29) as recorded in the Swedish National Patient Register. For this study, the coverage for all inpatient admissions was complete over the follow-up period and for outpatient admissions from 2001.^14,15^

### Exposure Variables

We investigated whether the number of moves over discrete periods of the life course and the cumulative distances moved were associated with subsequent psychosis risk. Age periods (0-6 years, 7-15 years, 16-19 years, and 20-29 years) were determined a priori to coincide with the transition through the Swedish public education system. We estimated the number of moves from the Total Population Register, which records the residential location of all people each year to one of 9200 “Small Area for Market Statistics” (SAMS) areas (median population size in 2011, 726 [interquartile range, 312-1378]). Small Area for Market Statistics are designed to be internally socioeconomically homogenous but differ according to the characteristics of the social environment, including deprivation and population density.^16^ For each participant and age period, we calculated the total number of times a change in SAMS residence occurred from year to year as: no moves (reference), 1 move, 2 moves, 3 moves, or 4 or more moves (eMethods in the Supplement). For each age period, we also estimated the cumulative distance moved by each participant (in kilometers) (eMethods in the Supplement).

### Covariates

We included confounder data on continuous age; sex; parental migration status (both parents Swedish-born vs at least 1 parent being foreign-born); biological parental history of severe mental illness (SMI), including nonaffective psychosis and bipolar disorders or mania with or without psychotic symptoms; the biological mother’s age at participant birth (as a proxy for paternal age); parental (biological or adoptive) death in any age period before the participant’s 16th birthday; SAMS population density at birth (eFigure 1 in the Supplement); participant compulsory school educational attainment; family disposable income at cohort entry; and university attendance (yes or no) (eMethods in the Supplement).

### Statistical Methods

We fitted discrete time proportional hazards models using complementary log-log models on the attained age scale (eMethods in the Supplement). Modeling proceeded as follows: (1) we modeled the crude association of the number of residential moves and cumulative distance moved in each age period with psychosis risk, (2) we added all the covariates to these models (adjustment 1) except for educational attainment at age 15 to 16 years (see Results), (3) we adjusted the models for residential move data in previous age periods (adjustment 2), and (4) because educational attainment at age 15 to 16 years may have been on the causal pathway between earlier moves and future psychosis risk, we restricted the adjustment for this variable to models of residential moves made after age 16 years (adjustment 3). When modeling residential moves after age 20 years, we excluded participants who had not reached this age by the end of the follow-up period or who were otherwise censored between age 16 to 19 years(n = 441 416; 30.1%). We included university attendance as a potential confounder (adjustment 4) and effect modifier of the association between residential moves and nonaffective psychosis risk in adulthood. To examine possible threshold effects in the geographical distances of residential moves, we inspected nonlinear distance functions using an inverse power (square root) transformation and compared this with a model that was fitted with a linear distance function via an inspection of Akaike Information Criterion scores in which lower scores indicated a better fit. We predicted and graphed marginal hazards over the cumulative distance moved in each age period. In a subgroup analysis, we investigated whether any associations of the geographical distances of residential moves with nonaffective psychosis risk were upheld among those who moved only once in each age period compared with those who never moved, with moving distance categorized as never moved, less than 5 km, 5 to 30 km, 30 to 100 km, 100 to 500 km, and 500 or more km. We reported hazard ratios (HRs) and 95% confidence intervals. Statistical significance was set at *P* < .05.

## Results

### Sample Characteristics

**Figure 1.**
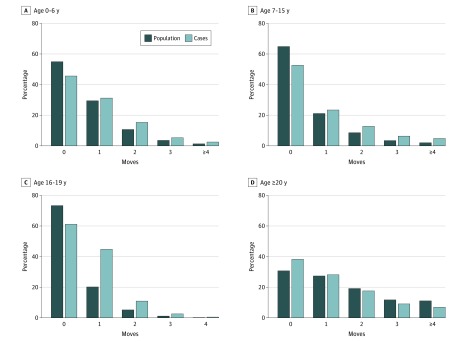
Residential Mobility by Number of Moves and Outcome Status by Age Period At ages 0 to 6 (A), 7 to 15 (B), and 16 to 19 years (C) the proportion of cases who moved once or more was greater than in the population at risk. By contrast, at 20 years and older (D), case participants were more likely to have never moved than the population at risk. Percentages in the 20 years or older group were estimated from participants who were not censored before this age (n = 998 967 [69.9%]). Percentages at all other age ranges were based on the full sample (N = 1 440 383). At age 16 to 19 years, the maximum number of possible moves during this period is 4.

Of 1 440 383 included participants (97.8% of cohort; eTable 1 and eResults in the Supplement), 4537 (0.31%; 95% CI, 0.30-0.33) received an *ICD-10* diagnosis of nonaffective psychotic disorder in Sweden during the follow-up period. The median age when receiving the first diagnosis was 20.9 years (interquartile range, 19.0-23.3). Participants with nonaffective psychosis were more likely to be men, come from a lower-income quintile, and have a foreign background, parental history of SMI, death of a parent before age 16 years, and lower educational attainment and less likelihood of attending university than the remainder of the cohort (Table 1). The distribution of the number of residential moves (Figure 1) and the cumulative distance moved differed for participants with nonaffective psychosis compared with the remainder of the cohort (Table 1). Thus, before age 20 years, case participants were more likely to have moved at least once and have had a longer cumulative distance moved (all *P* < .001); this pattern was reversed after age 20 years. The correlations within and between the number of moves and the cumulative distance moved were moderate (eTable 2 and eResults in the Supplement).

**Table 1.** Cohort Characteristics by Outcome Status

Variable	Outcome Status (Nonaffective Psychotic Disorder)		χ^2^	*df*	*P* Value
	Yes	No			
Participants (%)^a^	4537 (0.31)	1 435 846 (99.69)	NA	NA	NA
Median age (IQR)	20.9 (19.0-23.3)	22.4 (19.4-25.8)	21.6	NA	<.001^b^
Men (%)	2746 (60.5)	737 105 (51.3)	152.9	1	<.001
Foreign background (%)	851 (18.8)	181 462 (12.6)	153.2	1	<.001
Death of a parent before age 16 y (%)	173 (3.8)	32 279 (2.3)	50.3	1	<.001
Parental history of SMI (%)	467 (10.3)	42 695 (3.0)	833.6	1	<.001
Median maternal age at birth (IQR)	28.2 (24.4-32.3)	28.3 (24.9-32.0)	1.5		.12^b^
Income quintile (%)					
Highest	67 (1.5)	307 727 (2.1)	244.4	4	<.001
2	128 (2.8)	55 056 (3.8)			
3	305 (6.7)	139 489 (9.7)			
4	783 (17.3)	343 889 (24.0)			
Lowest	3254 (71.7)	866 685 (60.4)			
Population density at birth (pp km^2^)	1255.9 (158.7-3889.6)	739.3 (48.7-2850.6)	−13.3	NA	<.001^b^
Educational attainment at age 16 (%)					
Fail	650 (14.3)	64 934 (4.5)	2566.1	4	<.001
D or E grades	2622 (57.8)	863 511 (60.1)			
C grade	484 (10.7)	285 199 (19.9)			
A or B grades	198 (4.4)	173 691 (12.1)			
Missing	583 (12.9)	48 511 (3.4)			
University attendance^c^					
No	2312 (82.7)	621 033 (62.3)	499.3	1	<.001
Yes	483 (17.3)	375 139 (37.7)			
Moved ≥1 times (%), y^d^					
0-6	2467 (54.4)	646 645 (45.0)	159.3	1	<.001
7-15	2148 (47.3)	504 245 (35.1)	295.8	1	<.001
16-19	1762 (38.8)	382 908 (26.7)	342.1	1	<.001
20-29^c^	1726 (61.8)	690 316 (69.3)	74.5	1	<.001
Median cumulative distance moved (km) (10-90th percentile), y^e^					
0-6	1.2 (0-133.5)	0 (0-45.5)	−14.4	NA	<.001^b^
7-15	0 (0-86.3)	0 (0-27.4)	−19.0	NA	<.001^b^
16-19	0 (0-64.7)	0 (0-23.3)	−18.7	NA	<.001^b^
20-29^c^	0 (0-332.6)	0 (0-416.8)	9.9	NA	<.001^b^

Abbreviations: *df*, degrees of freedom; IQR, interquartile range; NA, not applicable; SMI, severe mental illness.

^a^ Row percentage.

^b^ Mann-Whitney *U* test for nonnormally distributed data.

^c^ Among those who did not exit the cohort before age 20 years (n = 998 967 [69.4%]).

^d^ For descriptive purposes, the number and proportion of people who moved 1 or more times in each period are displayed. A categorical variable (0,1,2,3, ≥4) was used for modeling purposes.

^e^ The 10th-90th percentile is reported in favor of the interquartile range, given substantial skew in the distribution of the exposures.

### Association Between Residential Mobility and Nonaffective Psychosis

We observed dose-response associations between greater moves at age 0 to 6 years, 7 to 15 years, and 16 to 19 years and the risk of nonaffective psychotic disorder in unadjusted survival models (Table 2) that persisted after adjusting for covariates (adjustment 1), including moves at previous ages (adjustment 2). Thus, compared with never moving, 1, 2, 3, or 4 or more moves between birth and age 6 years were associated with HRs of 1.13, 1.47, 1.46, and 1.83, respectively (adjustment 1; all *P* < .001) (Table 2). We observed similar associations for moves between age 7 to 15 years and stronger associations at age 16 to 19 years (adjustment 2; Table 2), with those moving in each year of this period having a 2.88-fold (95% CI, 1.89-4.40) increased risk compared with those who never moved. Further adjustment for educational attainment at age 15 to 16 years attenuated risks between age 16 to 19 years (adjustment 3, Table 2), but strong dose-response patterns remained (ie, moving 4 times; HR, 1.99; 95% CI, 1.30-3.05).

**Table 2.** Hazard Ratios (HR) for Nonaffective Psychosis by Number and Distance of Moves in Each Age Period

Exposures	Cases, No. (%)	HR (95% CI)				
		Crude	Adjustment 1^a^	Adjustment 2^b^	Adjustment 3^c^	Adjustment 4^d^
Age 0-6, y^e^						
1 move	1416 (31.2)	1.29 (1.21-1.38)	1.13 (1.04-1.21)	NA	NA	NA
2 moves	696 (15.3)	1.81 (1.66-1.97)	1.47 (1.32-1.62)	NA	NA	NA
3 moves	241 (5.4)	1.92 (1.68-2.20)	1.46 (1.25-1.70)	NA	NA	NA
≥4 moves	114 (2.5)	2.51 (2.08-3.03)	1.83 (1.48-2.26)	NA	NA	NA
Distance (square root)^f^	NA	1.37 (1.31-1.43)	1.13 (1.06-1.19)	NA	NA	NA
Age 7-15, y^e^						
1 move	1062 (23.4)	1.44 (1.34-1.55)	1.24 (1.15-1.34)	1.22 (1.13-1.32)	NA	NA
2 moves	580 (12.8)	1.94 (1.77-2.13)	1.58 (1.43-1.75)	1.51 (1.36-1.68)	NA	NA
3 moves	288 (6.4)	2.41 (2.13-2.72)	1.86 (1.62-2.13)	1.74 (1.52-2.01)	NA	NA
≥4 moves	218 (4.8)	2.99 (2.60-3.44)	2.14 (1.82-2.53)	1.95 (1.65-2.31)	NA	NA
Distance (square root)^f^	NA	1.52 (1.46-1.59)	1.16 (1.09-1.23)	1.11 (1.05-1.19)	NA	NA
Age 16-19, y^e^						
1 move	1125 (24.8)	1.45 (1.35-1.55)	1.47 (1.36-1.59)	1.35 (1.25-1.47)	1.28 (1.18-1.39)	NA
2 moves	497 (11.0)	2.47 (2.25-2.72)	2.45 (2.20-2.74)	2.08 (1.85-2.33)	1.79 (1.60-2.01)	NA
3 moves	117 (2.6)	2.61 (2.17-3.14)	2.55 (2.09-3.11)	2.00 (1.63-2.45)	1.57 (1.28-1.92)	NA
≥4 moves	23 (0.5)	3.96 (2.62-5.97)	3.87 (2.54-5.90)	2.88 (1.89-4.40)	1.99 (1.30-3.05)	NA
Distance (square root)^f^	NA	1.32 (1.26-1.40)	0.98 (0.92-1.06)	0.95 (0.88-1.02)	0.99 (0.98-1.03)	NA
Age ≥20, y^e^^,^^g^						
1 move	787 (28.2)	0.82 (0.74-0.89)	1.11 (1.01-1.22)	1.04 (0.94-1.14)	1.05 (0.96-1.16)	1.04 (0.94-1.14)
2 moves	491 (17.6)	0.69 (0.62-0.78)	1.18 (1.04-1.33)	1.05 (0.93-1.19)	1.07 (0.95-1.21)	1.05 (0.93-1.18)
3 moves	254 (9.1)	0.70 (0.61-0.81)	1.46 (1.25-1.71)	1.25 (1.07-1.47)	1.27 (1.08-1.49)	1.23 (1.05-1.44)
≥4 moves	194 (6.9)	0.88 (0.75-1.05)	2.35 (1.95-2.84)	1.91 (1.58-2.31)	1.91 (1.58-2.30)	1.82 (1.51-2.20)
Distance (square root)^f^	NA	0.60 (0.57-0.63)	0.58 (0.54-0.61)	0.56 (0.53-0.60)	0.60 (0.56-0.63)	0.67 (0.63-0.71)

Abbreviation: NA, not applicable.

^a^ Adjustment 1: age, quadratic age, sex, foreign background, parental history of severe mental illness, parental death before age 16 years, disposable income quintile, mother’s age at participant birth, population density at birth (log transformed people per square kilometer), and distance moved in age period.

^b^ Adjustment 2: adjustment 1 + number of and distance moved at previous ages.

^c^ Adjustment 3: adjustment 2 + educational attainment at age 15 to 16 years.

^d^ Adjustment 4: adjustment 3 + university attendance.

^e^ Reference group for number of moves: 0 moves.

^f^ A nonlinear distance function (square root transformation) was provided to better fit to the data than a linear term; assessed via Akaike Information Criterion (Figure 2; eTable 3 in the Supplement).

^g^ After age 20 years, model was restricted to cohort not censored before this point (n = 998 967).

There was weaker evidence that moving after age 20 years was associated with psychosis risk, with little variation in risk for those who moved fewer than 3 times in early adulthood, including after adjustment for educational attainment and university attendance (adjustment 4, Table 2). Nonetheless, those who moved more frequently (4 or more times) remained at a substantially elevated risk (HR, 1.82; 95% CI, 1.51-2.20). We found moderate evidence that this relative association was stronger in those who attended university (HR, 2.56; 95% CI, 1.55-3.54) than those who did not (HR, 1.65; 95% CI, 1.34-2.02; likelihood ratio test *P* = .02; eTable 3 in the Supplement), although marginal (ie, absolute) changes in the predicted probabilities of nonaffective psychosis for each additional move were similar in both groups (eFigure 2 in the Supplement).

**Figure 2.**
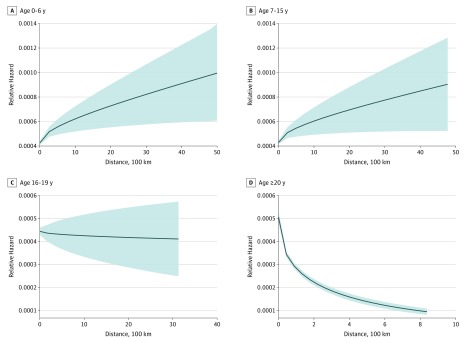
Predicted Hazard of Nonaffective Psychotic Disorder by Cumulative Distance Moved in Each Age Period Relative hazard of nonaffective psychotic disorder by cumulative distance between ages 0 to 6 (A), 7 to 15(B), 16 to 19 (C), and 20 or more years (D). Distances are displayed per 100 km up to a total of 1000 km. The shading denotes 95% CIs. Each model is based on the predicted relative hazard following modeling that was adjusted for the covariates listed in adjustment 2 (Table 2). Distances moved before age 16 years displayed a strong nonlinear trend, such that the relative hazard of nonaffective psychosis increased most quickly over shorter move distances (ie, within 30 km) before increasing at a slower rate over longer distances (with less certainty around point estimates). Distance moved between age 16 to 19 years was best modeled as a linear predictor, with no significant differences in the relative hazard of nonaffective psychosis observed by distance (Table 2). Cumulative distances moved after age 20 years were associated with a strong, nonlinear reduction in the relative hazard of nonaffective psychosis, particularly for moves up to approximately 30 km.

The cumulative distances moved at all ages were better modeled as nonlinear functions with respect to psychosis risk (eTable 4 in the Supplement). Independent of the number of moves, greater moving distances before age 16 years increased risk (Table 2), most sharply over shorter (ie, less than 30 km) distances moved (Figure 2A and B). Between age 16 to 19 years, we observed no evidence of any statistically significant association with distance. After age 20 years, greater cumulative distances moved were associated with decreased psychosis risk, with similar evidence of threshold effects at shorter distances (Figure 2D). These patterns were replicated in subgroup analyses that were restricted to participants who moved only once during each period compared with those who never moved (Table 3).

**Table 3.** Risk of Developing Nonaffective Psychosis by Distance Moved Among Those Who Moved Once vs Never Having Moved in Each Age Period

Distance Moved	Adjusted HR^a^ (95% CI)
Age 0-6, y	
No moves	1 [Reference]
<5 km	1.11 (1.01-1.22)
5-29 km	1.14 (1.03-1.26)
30-99 km	1.47 (1.25-1.74)
100-499 km	1.37 (1.04-1.50)
>500 km	1.58 (1.08-2.34)
Age 7-15, y	
No moves	1 [Reference]
<5 km	1.16 (1.05-1.28)
5-29 km	1.27 (1.14-1.42)
30-99 km	1.61 (1.32-1.95)
100-499 km	1.37 (1.10-1.70)
>500 km	1.58 (0.99-2.53)
Age, 16-19, y	
No moves	1 [Reference]
<5 km	1.10 (0.99-1.23)
5-29 km	1.10 (0.99-1.22)
30-99 km	1.09 (0.92-1.30)
100-499 km	1.03 (0.87-1.22)
>500 km	0.82 (0.53-1.27)
Age, ≥20, y	
No moves	1 [Reference]
<5 km	0.41 (0.36-0.46)
5-29 km	0.24 (0.21-0.27)
30-99 km	0.20 (0.16-0.24)
100-499 km	0.16 (0.13-0.19)
>500 km	0.08 (0.05-0.12)

Abbreviation: HR, hazard ratio.

^a^ Adjusted for age, quadratic age, sex, foreign background, family history of severe mental illness, parental death before age 16 years, disposable income quintile, mother’s age at participant birth, population density at birth (log transformed people per square kilometer,) and distance and number of moves (categorical) in previous age periods (except age 0-6 years).

In a sensitivity analysis (eTable 5 in the Supplement), we presented results from a fully mutually adjusted model of the number and distances of moves at each period of the life course to facilitate comparability with earlier studies.^4^ In this model, the association between the number of moves and psychosis risk was most substantially attenuated at ages 0 to 6 years and 7 to 15 years; the number of moves between age 16 to 19 years continued to exhibit a dose-response association with later psychosis risk.

## Discussion

In this study we show that greater residential mobility during childhood and adolescence is associated with a dose-response increase in risk of developing nonaffective psychosis. These patterns were impervious to adjustment for psychiatric family history and sociodemographic indicators, including family disposable income, and could not be explained by moves at previous ages nor, when relevant, educational attainment at age 15 to 16 years or university attendance. The larger effect sizes for moves between age 16 to 19 years is consistent with the thesis that residential mobility is associated with nonaffective psychosis through a mechanism that is at its most sensitive during adolescence, in line with earlier observations.^4^ We also found that longer geographical distances of residential moves during childhood and early adolescence were associated with increased risk, independently of the number of moves, particularly for moves more than approximately 30 km, which was consistent with the distances at which the disruption of school-based or other social networks were more likely.

The association between residential mobility and nonaffective psychosis was different in young adults. While there was some evidence that moving frequently (3 or more times) between age 20 to 29 years was associated with increased risk, no differences emerged for individuals who moved fewer times in early adulthood. Moreover, moving longer distances in adulthood was strongly associated with a reduced risk of nonaffective psychosis. Taken together, these results suggest that residential stability in early life and some geographical mobility in adulthood do not increase and may confer protection against psychosis risk.

### Potential Mechanisms

The most supported explanation as to how residential mobility could have an association with nonaffective psychosis is that a change of residence disrupts an individual’s ability to form and maintain friendships or fit within a peer group. Social isolation is likely to increase one’s vulnerability to the effects of life stressors. For example, exposure to stressful life events could have a greater impact on negative schemata, low self-esteem, and cognitive biases that are associated with psychosis^17,18,19^ without the buffering effect of stable friendships.

Some studies suggest that part of the association of residential mobility with psychosis is mediated via having to change schools, and that loss of peer relationships and increased social isolation may be involved in the pathway to risk.^20,21^ Residential mobility may disrupt social relationships and be associated with subsequent psychosis risk if it necessitates a change in schools, and if it occurs at a time when relationships with peers become as or more important than family-based ones. Our finding that the greatest risk was observed for residential moves during late adolescence, independent of academic ability at age 15 to 16 years, is consistent with this thesis, as is our finding that longer moves predicted greater psychosis risk. Nonetheless, not all studies have observed associations between school mobility and psychosis risk,^22^ suggesting that beyond the school context, other peer group relationships, including family, kinship, and wider neighborhood ties, may also be relevant.

We have previously shown that the characteristics that mark someone out as different from most of their peers, whether at a school level or neighborhood level, are associated with an increased risk of psychosis,^7,14^ findings that are often conceptualized within the concept of social defeat. It has been hypothesized that social defeat contributes causally to psychosis risk via the sensitization of the mesolimbic dopamine system,^19^ the disruption of which is a widely supported biological theory of schizophrenia.^23^ Support for this theory is evident from animal model studies,^24,25^ and such a mechanism might explain how greater residential mobility, especially during adolescence, increases psychosis risk if it is subsequently accompanied by changes in the propensity to experience social adversities, such as social isolation and/or exposure to stressful life events.^26^ It is also possible that the association between residential mobility and psychosis is, at least in part, mediated by factors other than disrupted social relationships (eg, an earlier initiation of drug use^27,28,29^ or reduced engagement with health, social, and education services^30,31,32^).

Our findings with respect to early adulthood somewhat contrast those for mobility at earlier ages. Moving once or twice during this period did not alter risk, and those who moved longer distances were substantially less likely to subsequently develop psychotic disorder; cumulative distances moved accounted for the change in the direction of the unadjusted, protective association between the number of moves and nonaffective psychosis risk to a risk factor in adjusted models (data available from the authors). These findings were not substantially confounded by university attendance. Changing residence after age 20 years, the age at which students in Sweden complete their university entrance examinations, is likely to reflect the onset of independence for an individual, be it through university attendance or entry into the labor market, and may explain why there were weaker associations with the number of moves and a strong negative association between moving distance and psychosis risk during this period. Although less consistently than for greater cognitive ability,^33,34^ higher levels of education has been associated with reduced risk of nonaffective psychosis,^35,36^ Nonetheless, frequent moves (particularly 4 or more) in early adulthood remained associated with increased psychosis risk irrespective of university attendance, and we hypothesize that this reflects the more chaotic lifestyle of individuals who are at higher risk of developing psychosis (eg, as a result of substance misuse or the presence of financial, social, or other mental health difficulties).

### Strengths and Limitations

This study has several strengths, including the longitudinal design and large sample size that is highly representative of the entire (Swedish-born) population. The prospective measurement of our exposure and the use of register data eliminate the possibility of recall bias. Our outcome measure is known to have good concurrent validity for diagnoses of schizophrenia in this register,^37,38^ and psychiatric care in Sweden is both accessible and free. Using geographical information systems data to estimate the number and distance of small area moves over the life course is a further strength of this study, although we acknowledge that the distances were based on “as the crow flies” estimates. We controlled for several confounders that may have precipitated residential mobility, including parental death, a parental history of SMI, urban birth, income, younger maternal age at birth, and, with respect to moves made after age 16 years, educational attainment. We acknowledge that we did not have data on other potential confounders, including other adverse childhood experiences such as family discord or parental separation. Nor did we have data on measures such as quality of friendships and peer problems, such as bullying, to test potential mediating pathways. We also lacked direct data on school changes as an index of disruption to peer relationships. Selection bias might be present from the small amount of incomplete geographical data in this study (eTable 1 in the Supplement), although this might be expected to have underestimated our associations, given that reasons for missingness include homelessness and being in prison, which are further markers of residential mobility and are associated with nonaffective psychosis. While reverse causation is unlikely to explain our findings, it remains feasible that subthreshold or prodromal symptoms during childhood or early adolescence led some families to change residence in the hope that a different school, neighborhood, or proximity to specialist health care clinicians might improve their child’s well-being. Finally, in other studies, residential mobility has been associated with bipolar disorder and substance use disorders, suggesting that residential mobility may be a nonspecific risk factor for several psychiatric disorders.^4,5^

## Conclusions

Accumulating evidence supports childhood and adolescent residential mobility as an independent risk factor for psychosis and other mental health outcomes. Efforts are now required to examine the reasons for this, which may include precipitating factors such as family discord, as well as the effect such moves are likely to have on peer group formation and social support during critical periods of development; these findings will also have implications for informing the development of child health services and social policy. It is important that health, social, and educational practitioners ensure that children and adolescents who are newly resident to their neighborhoods receive adequate support to minimize the risks of adverse outcomes during adulthood, and every effort should be made to ensure the effective transfer of care for highly mobile children who are already in contact with health and social services.

## Notes

Supplement. eMethods. eResults. eFigure 1. Positively skewed distribution of population density at birth in cohort eFigure 2. Predicted probability of non-affective psychosis by number of moves aged 20 years and older, by university attandence eTable 1. Cohort characteristics by missing data type eTable 2. Correlation between number and cumulative distance of moves at different age periods eTable 3. Effect modification of number of moves after 20 years old on non-affective psychosis risk, by university attendance eTable 4. Model fit comparisons for linear versus non-linear distance functions eTable 5. Risk of non-affective psychoses after mutual adjustment for moves in all periods
