# Supplementary material for: Association of Residential Mobility Over the Life Course With Nonaffective Psychosis in 1.4 Million Young People in Sweden
Source: JAMA Psychiatry. 2018 Aug 22;75(11):1128–36. doi: 10.1001/jamapsychiatry.2018.2233 (PMC6248097; doi:10.1001/jamapsychiatry.2018.2233)
Supplement: Supplement. — eMethods. eResults. eFigure 1. Positively skewed distribution of population density at birth in cohort eFigure 2. Predicted probability of non-affective psychosis by number of moves aged 20 years and older, by university attandence eTable 1. Cohort characteristics by missing data type eTable 2. Correlation between number and cumulative distance of moves at different age periods eTable 3. Effect modification of number of moves after 20 years old on non-affective psychosis risk, by university attendance eTable 4. Model fit comparisons for linear versus non-linear distance functions eTable 5. Risk of non-affective psychoses after mutual adjustment for moves in all periods [file jamapsychiatry-75-1128-s001.pdf]

Supplementary Online Content

Price C, Dalman C, Zammitt S, Kirkbride JB. Association of residential mobility over the life course with nonaffective psychosis in 1.4 million young people in Sweden. *JAMA Psychiatry*. Published online August 22, 2018.
doi:10.1001/jamapsychiatry.2018.2233

eMethods.
eResults.
eFigure 1. Positively skewed distribution of population density at birth in cohort
eFigure 2. Predicted probability of non-affective psychosis by number of moves aged 20 years and older, by university attandence
eTable 1. Cohort characteristics by missing data type
eTable 2. Correlation between number and cumulative distance of moves at different age periods
eTable 3. Effect modification of number of moves after 20 years old on non-affective psychosis risk, by university attendance
eTable 4. Model fit comparisons for linear versus non-linear distance functions
eTable 5. Risk of non-affective psychoses after mutual adjustment for moves in all periods

This supplementary material has been provided by the authors to give readers additional information about their work.

© 2018 Price C et al. *JAMA Psychiatry*.

e*Methods*

Sample

From our initial cohort (N=1,472,446) we excluded 30,368 (2.1%) participants partially or wholly missing geographical data on residential location, and 1,695 (0.1%) participants missing covariate information, leaving a final sample of 1,440,383 participants with complete residential mobility data.

Further exposure variable information

All people are required to inform Statistics Sweden of any residential move by law. Residential moves can be detected in the Swedish Register of the Total Population (RTP) by comparing a participant's SAMS area from one year to the next. By law, people must notify Statistics Sweden when they move residences, and changes in SAMS area are recorded annually in the Register of the Total Population. This methodology makes it possible to detect moves between small areas (SAMS) across the life course, but does not capture multiple moves within a given year, or moves within the same SAMS area. For each participant in each age period (0-6, 7-15, 16-19, 20+ years), we also estimated cumulative distance moved by each participant (in kilometers [km). To do so, we constructed a matrix of the Euclidian distance between any two SAMS areas in ArcGis (v10.4), based on their centroid XY coordinates, giving rise to a look-up table of 84.6m possible inter-SAMS moves.

Covariate information

Parental information was obtained from linkage between the Multigenerational register and other registers of interest (i.e. RTP, National Patient Register). Maternal age was strongly correlated with paternal age (corr=0.69), but had less missing data (0.09% vs. 0.56%), and so was preferred. Parental history of severe mental illness [SMI] was coded as a binary variable for either biological parent ever having been diagnosed with non-affective psychosis, bipolar disorder with psychosis or bipolar disorder without psychosis since 1969, when the National Patient Register began (ICD-9 codes: 295.x, 296.x; ICD-10 codes: F20-31). Parental death before the participant's 16th birthday was also defined as a binary variable, and included either the biological or adoptive parents. Population density (in people per square kilometer [ppkm2]) was estimated for all SAMS between 1982-1995 based on total population size divided by area. All participants were assigned their SAMS population density at birth. Due to substantial positive skew (eFigure 1), a continuous log transformed

© 2018 Price C et al. *JAMA Psychiatry*.

variable was used in analyses to control for this confounder. Participant educational attainment may have been associated with propensity to move during the follow-up period[31] and is known to be related to psychosis risk.[35] To control for this confounder, we obtained educational attainment upon leaving compulsory school at age 15/16 years from the National School Register (see MacCabe et al[35] for full details). In the year students turn 16 years old, they are typically examined on 16 compulsory subjects. Those examined on 7 or more subject areas are given an overall grade point average (GPA). GPAs greater than or equal to 10 are converted to an A to E classification, with GPAs less than 10 recorded as failing compulsory school. Here, we grouped participants into the following categories of compulsory school educational attainment: fail (which included those with a GPA<10, and those examined on less than 7 subjects), grades D & E, grade C (the modal grade), grades A & B, and those missing data on educational attainment, We retained those with missing data on educational attainment in the analysis as a separate category because this may have indicated they did not attend or complete compulsory schooling in Sweden. Family disposable income was calculated based on total family income from all registered sources, including wages, welfare benefits, other social subsidies, and pensions, according to the Longitudinal Integration Database for Health Insurance and Labour (LISA). Family disposable income was categorized into quintiles, relative to all other people in Sweden in the given year to account for inflation. University attendance, as recorded in the LISA (denoted by codes 4XX to 6XX), was coded as a binary variable for analyses restricted to participants with follow-up data after age 20 years old.

Statistical analyses

We fitted discrete time proportional hazards models to estimate the effect of residential moves during childhood adolescence and early adulthood on risk of non-affective psychotic disorders Discrete time survival analysis was necessary because our exposure (number of times moved) was only made available by Statistics Sweden for discrete intervals (years; see above). To do so, we constructed our cohort as a person-period dataset on the attained-age scale, such that each year of attained age was modelled as a separate observation within participants, with the outcome of interest (diagnosis of non-affective psychosis) coded as 1 in the year of exit, or 0 otherwise. Given strong evidence of non-linear associations between age and psychosis risk,[37,38] we modelled and controlled for attained age as a function of linear and quadratic terms. Total number of moves before cohort entry (0-6, 7-15 years), cumulative distances moved in each period and confounders

© 2018 Price C et al. *JAMA Psychiatry*.

were modelled as time-fixed covariates (invariant across levels of attained age); number of moves during the follow-up period (16-19, 20-29 years) were modelled as time-varying covariates.

eResults

Missing data

Participants excluded due to missing data did not differ from included participants in terms of the proportion diagnosed with non-affective psychosis (see eTable 1). They were, however, more likely to be younger and have a foreign background than included participants. Participants excluded due to missing geographical data (30,368 (2.1%)) were also more likely to have a parental history of non-affective psychosis, be women, belong to the highest or lowest income quintiles and have an older mother at birth, but were less likely to have experienced death of a parent during childhood. Those missing covariate data (N=1,695; 0.1%) were more likely to have moved at least once between 0-6 and 7-15 years old and come from higher income quintiles, but less likely to have moved after 16 years than those included in the study; these differences were also reflected in cumulative distances moved.

Correlations between number of and distances moved within and between age periods

Correlations ranged from 0.02-0.32 for number of moves in one age period to another, to 0.04-0.20 for cumulative distance moved in one age period to another, and 0.39-0.54 for within-period correlations between number of moves and distance moved (see eTable 2).

© 2018 Price C et al. *JAMA Psychiatry*.

eFigure 1: Positively skewed distribution of population density at birth in cohort[1]

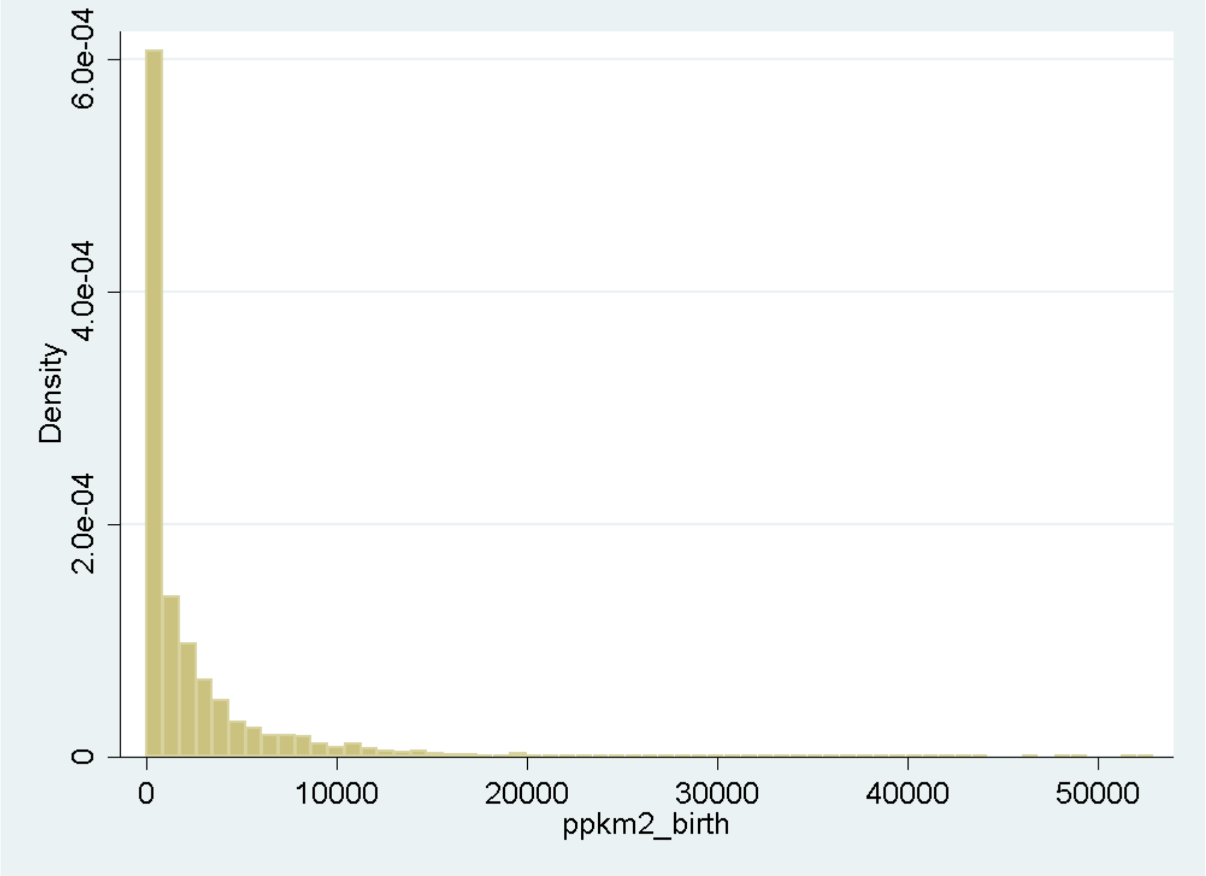

[1]Population density at birth was estimated at the SAMS geographical area in people per square kilometer (ppkm2)

eFigure 2: Predicted probability of non-affective psychosis by number of moves aged 20 years and older, by university attandence[1]

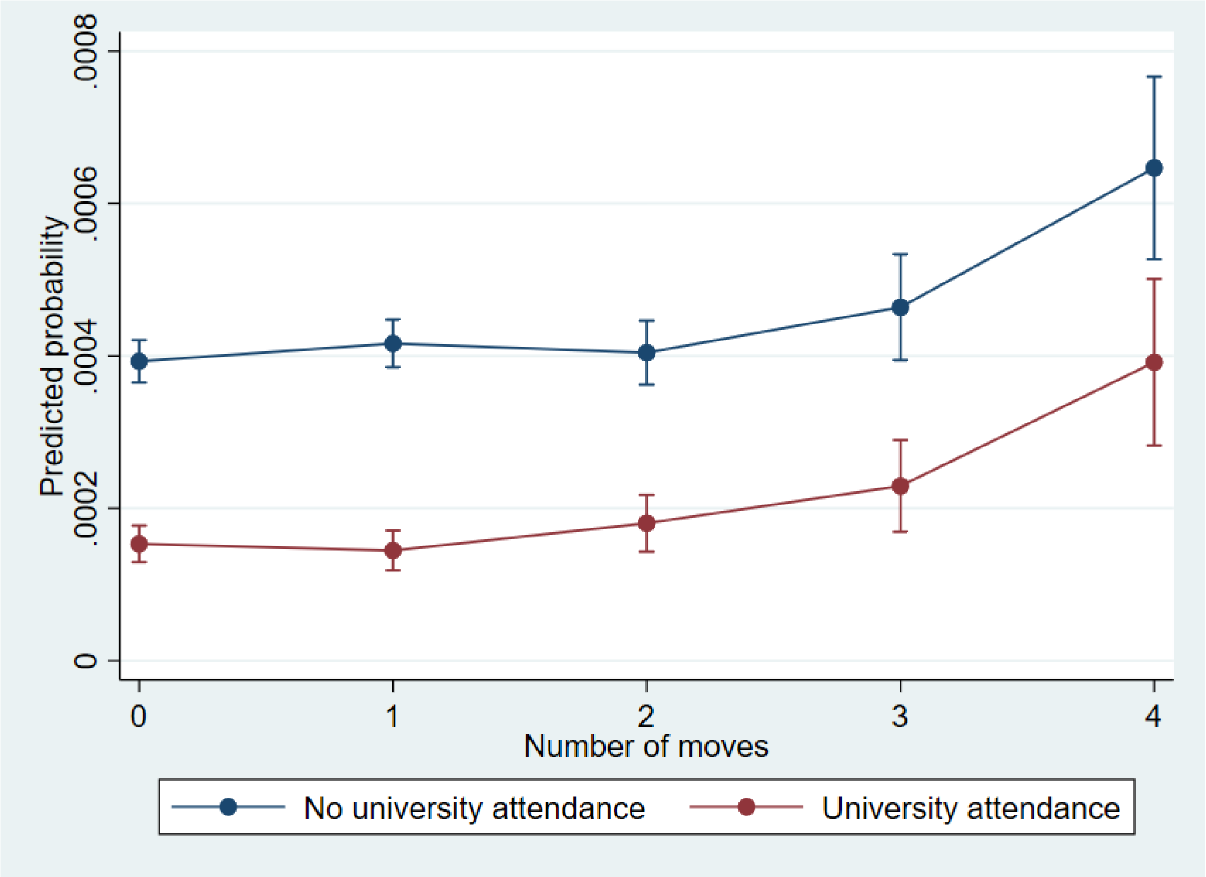

Legend: Predicted probabilities obtained from the model presented in eTable 3, restricted to the cohort not censored prior to their 20th birthday. Although *relative risks* for increased number of moves in adulthood on psychosis appeared to be stronger in those attending university compared with those not attending university (eTable 3), *absolute* predicted probabilities were higher in non-university attendees across all number of moves. Moving 4 or more times led to a similar absolute increase in the predicted probability of non-affective psychotic disorder in both groups.

© 2018 Price C et al. *JAMA Psychiatry*.

eTable 1. Cohort characteristics by missing data type

	Missing type[a]					
**Variable**	**SAMS data**		**X^2 (*df*); p-value**	**Covariate information**		**X^2 (*df*); p-value**
Participants (%)[b]	30,368	(2.1)	-	1,695	(0.1)	-
Non-affective psychosis (%)	112	(0.4)	2.7 (1); p=0.10	7	(0.4)	0.5 (1); p=0.47
Median age (IQR)	21.3	(18.8-24.5)	34.8; p<0.001[c]	19.7	(16.6-24.5)	19.4; p<0.001[c]
Men (%)	15,420	(50.8)	4.1 (1); p=0.04	856	(50.5)	0.5 (1); p=0.48
Foreign background (%)	15,141	(49.9)	$3.5x10^4$ (1); p<0.001	1,462	(86.3)	8246.7 (1); p<0.001
Death of a parent before 16 years (%)	481	(1.8)	23.9 (1); p<0.001	40	(2.4)	0.1 (1); p=0.77
Parental history of SMI (%)	960	(3.2)	2.8 (1); p=0.10	36	(2.1)	4.4 (1); p=0.04
Median maternal age at birth (IQR)	28.7	(25.1-32.1)	-6.0; p<0.001[c]	28.0	(24.3-31.8)	1.8; p=0.07[c]
Income quintile			2898.5 (1); p<0.001			9.8. (4); p=0.04
Highest	1,466	(5.0)		32	(2.5)	
2	1,889	(7.4)		41	(3.2)	
3	2,626	(9.0)		136	(10.7)	
4	4,123	(14.1)		265	(20.8)	
Lowest	19,222	(65.8)		802	(62.9)	
Moved 1+ times (%)[d]						
0-6 years	-			882	(52.0)	33.2 (1); p<0.001
7-15 years	-			661	(39.0)	10.9 (1); p=0.001
16-19 years	-			337	(19.9)	40.3 (1); p<0.001
20-29 years[e]	-			524	(64.4)	9.2 (1); p=0.002
Median cumulative distance moved (km) (10-90th centile)[f]						
0-6 years	-			0.0	(0.0-151.4)	-5.0; p<0.001[c]
7-15 years	-			0.0	(0.0-47.9)	-3.1; p=0.002[c]
16-19 years	-			0.0	(0.0-10.9)	6.6; p<0.001[c]
20-29 years[e]	-			9.5	(0.0-80.0)	2.2; p=0.03[c]

IQR: Inter-quartile range; *df*: degrees of freedom; X^2: Chi^2; SMI: severe mental illness
[a]Either missing SAMS geographical data during observation or missing covariate information
[b]Proportion with missing data from initial cohort of 1,472,446
[c]Mann-Whitney u-test for non-normally distributed data
[d]For descriptive purposes, the number & proportion of people who moved 1+ times in each period are displayed. A categorical variable (0,1,2,3,4+) was used for modelling purposes
[e]Amongst those who did not leave the cohort prior to 20 years old
[f]The 10th-90th percentile is reported in favor of the interquartile range, given substantial skew in the distribution of the exposures

eTable 2. Correlation between number and cumulative distance of moves at different age periods

	Variable[1]	1.	2.	3.	4.	5.	6.	7.	8.
**1.**	Number of moves, 0-6y	1							
**2.**	Number of moves, 7-15y	0.322*	1						
**3.**	Number of moves, 16-19y	0.176*	0.286*	1					
**4.**	Number of moves, 20+y	0.021*	0.034*	0.154*	1				
**5.**	Distance moved, 0-6y	0.399*	0.140*	0.071*	0.034*	1			
**6.**	Distance moved, 7-15y	0.155*	0.397*	0.124*	0.028*	0.205*	1		
**7.**	Distance moved, 16-19y	0.063*	0.096*	0.391*	0.092*	0.080*	0.149*	1	
**8.**	Distance moved, 20+y	-0.003*	-0.010*	0.054*	0.537*	0.054*	0.041*	0.139*	1

[1]y is years. Distance moved is cumulative distance moved in kilometers

*p<0.01; due to large sample all correlations met this statistical significance threshold. Shaded correlations denote most pertinent comparisons to the results of this paper

© 2018 Price C et al. *JAMA Psychiatry*.

eTable 3: Effect modification of number of moves after 20 years old on non-affective psychosis risk, by university attendance

Model[a]	No university attendance		University attendance	
	**HR**	**(95% CI)**	**HR**	**(95% CI)**
Number of moves, 20+ years				
No moves	Ref		Ref	
1	1.06	(0.96-1.17)	0.94	(0.75-1.19)
2	1.03	(0.90-1.17)	1.17	(0.91-1.52)
3	1.18	(0.99-1.40)	1.50	(1.10-2.03)
4	1.65	(1.34-2.02)	2.56	(1.55-3.54)

[a]Model restricted to those not censored prior to 20 years old (N=988,974). Adjusted for age, quadratic age, sex, foreign background, family history of severe mental illness, parental death before 16 years, disposable income quintile, mother's age at participant birth, population density at birth, distance and number of moves (categorical) in previous age periods and educational attainment at 15/16 years. Statistical interaction was observed between number of moves aged 20 years and older and university attendance on non-affective psychosis risk (likelihood ratio test on 4 degrees of freedom: X^2: 11.3; p=0.02).

eTable 4: Model fit comparisons for linear versus non-linear distance functions

**Model**[a]	**Linear distance AIC**	**Square root distance AIC**
Cumulative distance moved between:		
Ages 0-6	76,878.2	76,874.8
Ages 7-15	76,651.9	76,650.1
Ages 16-19	76,455.6	76,453.4
Ages 20+	51,374.8	51,120.2
Single distance moved between[b]:		
Ages 0-6	59,895.8	59,885.3
Ages 7-15	59,439.8	59,422.9
Ages 16-19	66,782.6	66,782.7
Ages 20+	35,569.7	35,194.0

[a]Models Adjusted for age, quadratic age, sex, foreign background, family history of severe mental illness, parental death before 16 years, disposable income quintile, mother's age at participant birth, population density at birth, distance and number of moves (categorical) in previous age periods (except 0-6 years old) and number of moves in current period
[b]Restricted to those moving once or never in each period

© 2018 Price C et al. *JAMA Psychiatry*.

eTable 5. Risk of non-affective psychoses after mutual adjustment for moves in all periods

	Mutual adjustment[a]	
**Exposures**	**HR**	**(95% CI)**
**Age 0-6**		
0 moves	1	
1 move	0.99	(0.90-1.09)
2 moves	1.12	(0.99-1.28)
3 moves	0.98	(0.81-1.19)
4+ moves	1.04	(0.80-1.36)
Distance (square root)	1.16	(1.08-1.25)
**Age 7-15**		
0 moves	1	
1 move	1.10	(1.00-1.21)
2 moves	1.25	(1.10-1.42)
3 moves	1.17	(0.97-1.40)
4+ moves	1.22	(0.98-1.51)
Distance (square root)	1.14	(1.05-1.23)
**Age 16-19**		
0 moves	1	
1 move	1.10	(1.00-1.21)
2 moves	1.39	(1.21-1.60)
3 moves	1.35	(1.07-1.70)
4 moves	1.46	(0.89-2.40)
Distance (square root)	1.25	(1.14-1.36)
**Age 20 plus**		
0 moves	1	
1 move	1.05	(0.96-1.16)
2 moves	1.07	(0.95-1.21)
3 moves	1.27	(1.08-1.49)
4+ moves	1.90	(1.58-2.30)
Distance (square root)	0.60	(0.57-0.64)

[a]Cohort restricted to N=998,967 not censored prior to 20 years old. Model adjusted for all covariates listed in Adjustment 3 (see Table 2) i.e. including move data and educational attainment at later time points.

© 2018 Price C et al. *JAMA Psychiatry*.
